# Using automated voice messages linked to telephone counselling to increase post-menstrual regulation contraceptive uptake and continuation in Bangladesh: study protocol for a randomised controlled trial

**DOI:** 10.1186/s12889-017-4703-z

**Published:** 2017-10-03

**Authors:** Kate Reiss, Kathryn Andersen, Sharmani Barnard, Thoai D. Ngo, Kamal Biswas, Christopher Smith, James Carpenter, Kathryn Church, Sadid Nuremowla, Erin Pearson

**Affiliations:** 1 Department of Population Health, London School of Hygiene and Tropical Medicine, Keppel Street, London, WC1E 7HT UK; 2 Ipas, P.O. Box 9990, Chapel Hill, NC 27515 USA; 3 Centre for Global Health and Health Partnerships, School of Population Sciences and Health Services Research, King’s College London, Room 2.63 Weston Education Centre, Cutcombe Road, London, SE5 9RJ UK; 4 Population Council, One Dag Hammarskjold Plaza, New York, NY 10017 USA; 5 Ipas Bangladesh, Eureka Saleha Palace, (Flat - B2 & C2), 2nd Floor, House #2F-1-3, Mymensingh Road, Shahbag, Dhaka, 1000 Bangladesh; 6 Department of Medical Statistics, London School of Hygiene and Tropical Medicine, Keppel Street, London, WC1E 7HT UK; 7 MRC Clinical Trials Unit at UCL, Aviation House, Kingsway, London, UK; 8 Evidence to Action Team, Health Systems Department, Marie Stopes International, London, W1T 6LP UK; 9 Independent Consultant, Dhaka, Bangladesh; 10 Harvard T.H. Chan School of Public Health, 677 Huntington Avenue, Boston, MA 02115 USA

**Keywords:** mHealth, Mobile phone, Post-menstrual regulation, Abortion, Contraception, Family planning, Bangladesh, Randomised controlled trial, Protocol

## Abstract

**Background:**

Adoption of modern contraceptive methods after menstrual regulation (MR) is thought to reduce subsequent unwanted pregnancy and abortion. Long-acting reversible contraceptives (LARCs) are highly effective at reducing unintended pregnancy, but uptake in Bangladesh is low. Providing information on the most effective methods of contraception increases uptake of more effective methods. This protocol describes a randomised controlled trial of an intervention delivered by mobile phone designed to support post-MR contraceptive use in Bangladesh.

**Methods:**

This is a multi-site single blind individual randomised controlled trial. At least 960 women undergoing MR procedures at selected facilities will be recruited after their procedure by female research assistants. Women will be randomised into the control or intervention group with a 1:1 ratio.

All participants will receive usual clinic care, including contraceptive counselling and the telephone number of a non-toll-free call centre which provides counselling on MR and contraception. During the 4 months after their MR procedure, intervention participants will be sent 11 recorded interactive voice messages to their mobile phone about contraception with a focus on their chosen method and LARCs. Each message allows the participant to connect directly to the call centre. The intervention is free to the user. The control group will receive no messages delivered by mobile phone. All participants will be asked to complete an in-person questionnaire at recruitment and follow-up questionnaires by telephone at 2 weeks, 4 months and 12 months after their MR. The primary outcome for the trial will be self-reported LARC use 4 months post-MR. Secondary outcomes include LARC use at 2 weeks and 12 months post-MR, use of any effective modern contraceptive method at 2 weeks, 4 months and 12 months post-MR, and contraceptive discontinuation, contraceptive method switching, pregnancy, subsequent MR and experience of violence during the 12 month study period.

**Discussion:**

Mobile phones offer a low-cost mechanism for providing individualised support to women with contraception outside of the clinic setting. This study will provide information on the effects of such an intervention among MR clients in Bangladesh.

**Trial registration:**

Trial registered with clinicaltrials.gov Registration number: NCT02579785 Date of registration: 16th October 2015.

## Background

Abortion is illegal in Bangladesh except to save the life of a women, however ‘menstrual regulation’ (MR) is allowed as an “interim method to establish a state of non-pregnancy in a woman who is at risk of being pregnant” [1]. MR is legal when provided by a registered service provider as a surgical procedure with manual vacuum aspiration (MVA) up to 12 weeks from the last menstrual period or as a medical procedure (menstrual regulation with medication (MRM)) up to 9 weeks gestation using a combination regimen of mifepristone and misoprostol [2, 3]. Economic, cultural and informational barriers limit access to legal MR services and many women still turn to illegal abortions which are often unsafe, putting them at risk of harm [4–6]. MR and abortion incidence can be estimated using facility and population surveys, service numbers and key informant interviews [6, 7]. In 2014, an estimated 430,000 MR procedures took place at health facilities in Bangladesh [8].

Use of modern contraception has the potential to avert the majority of abortion related morbidity and mortality around the world [9, 10]. In Bangladesh, 12% of currently married women aged 15–49 years have an unmet need for family planning [11], this figure is slightly lower than the average for Southern Asia (14%) [12]. The type of contraceptives a woman uses has implications for unintended pregnancies: the contraceptive pill has a failure rate of 8% in the first year of typical use, whereas the most effective reversible methods of contraception, the intra-uterine device (IUD) and the implant (termed long-acting reversible contraceptives or LARCs), have failure rates of between 0.05% and 0.8% in the first year of use [13]. LARCs also have lower discontinuation rates and higher user satisfaction than short-term methods (such as the pill, condom, and injectable) [11, 13–15]. In Bangladesh, 40% of condom users and 34% of pill users discontinue their method within a year, the latter often due to side effects or health concerns; however, the rate of discontinuation for implant users is just 7% [11]. Despite this, short-term methods dominate in Bangladesh, in 2014 the method mix was as follows: 27.0% of married women of reproductive age used the pill, 12.4% used the injectable, 6.4% condoms, 1.7% the implant and 0.6% the IUD [11]. A further 8.4% of women rely on natural methods such as abstinence and withdrawal and 5.8% use sterilisation [11]. Interventions have demonstrated that removal of barriers to LARC use, such as knowledge gaps and cost, can increase uptake of these methods [14, 16].

Interventions are needed to support continuation and correct use of short-term methods and to address barriers to use of LARCs and permanent methods of contraception among Bangladeshi women at risk for unwanted pregnancy. MR clients are a key population to target as they often want to delay or limit future pregnancies. The WHO recommends provision of post-abortion family planning as an effective way of reducing subsequent unwanted pregnancy and abortion [17]. Most facilities in Bangladesh offer post-MR family planning counselling and services however gaps remain: the full range of contraceptive methods is not always available and counselling is often limited to the day of the procedure as WHO does not currently recommend routine follow-up for MR cases [17, 18].

The fast, cheap and confidential communication offered by mobile phones and their high and increasing prevalence of ownership in developing countries makes them a viable means of delivering sensitive information to large populations. In contrast to counselling on the day of an MR procedure, mobile-phone-based health (mHealth) interventions can give women time to consider their contraceptive options. At the time of procedure, women’s focus may be on the MR rather than on family planning; in Cambodia women have said they find it difficult to make a decision on the day of procedure [19]. Furthermore, women may want time to discuss their family planning choices with their husband/partner or other family members [19]. Use of mobile phones in Bangladesh is high and increasing; in 2014, 89% of all households reported having a mobile phone [11] making it a good context for mHealth interventions. However inequities remain; among 20–29 year olds, 90% of men but just 54% of women have a phone [11].

A feasibility study conducted in Bangladesh in 2013 among 120 public sector MR and post abortion care clients randomly assigned 60 participants to receive one-way short message service (SMS) messages reminding them about contraception (Biswas K, Hossain A, Chowdhury R, Andersen K, Sultana S, Shahidullah SM, Pearson E: Using mHealth to Support Postabortion Contraceptive Use: Results from a Feasibility Study in Urban Bangladesh, Unpublished). The intervention was well received: 76% of women in the intervention group reported they would sign up to the messages again and 93% said they felt the messages had helped them to use contraception. Participants also expressed an interest in speaking to a hotline counsellor, and half said they would prefer automated calls to SMS messages as it would be better for confidentiality.

Globally, some evaluations of mHealth interventions have demonstrated that they can be effective in improving health behaviours and outcomes, but the evidence is limited and outcomes are mixed [20, 21]. Findings suggest that multifaceted approaches may be more effective but further research is required to establish how to optimise interventions [20]. Evidence for the effectiveness of mHealth interventions aimed at improving contraceptive use is limited and shows mixed results [22]. One trial focused on post-abortion contraception and found that interactive voice messages linked to call centre counselling sent to abortion clients in Cambodia were successful in increasing contraceptive use at 4 months but not at 12 months [16].

This trial aims to examine the effects of an intervention delivered by mobile phone designed to support contraceptive use among public and private sector MR clients in three divisions of Bangladesh. The trial hypothesis is that the women who receive a multi-component mHealth intervention will have higher rate of LARC use at 2 weeks, 4 months and 12 months post-MR, compared to women who do not receive the mHealth intervention.

## Methods/design

### Trial design

The intervention will be evaluated using a multi-site, single-blinded RCT. Participants are randomized to the intervention (interactive voice messages that optionally link to telephone counselling in addition to standard care plus the call centre number) or control (standard care plus call centre number) with a 1:1 ratio.

### Study setting

Women will be recruited from Marie Stopes Bangladesh (MSB) facilities and public sector facilities that are supported by Ipas Bangladesh. MSB operates 140 clinics that offer sexual and reproductive health services including family planning, post-abortion care (PAC) and MR across the country. Ipas Bangladesh currently supports 82 government and non-government Reproductive Health Services Training and Education Programme (RHSTEP) facilities, providing training on woman-centred MR, PAC and post-abortion family planning for physicians and mid-level providers working in intervention clinics.

MSB and public-sector clinics supported by Ipas will be eligible to participate if they are in Dhaka, Chittagong or Sylhet Division, have an average monthly case load of at least 20 MR clients and have no other intervention studies underway. Eligible clinics will be stratified into primary, secondary and tertiary levels to ensure representation from all facility types as these facilities serve different populations of women. Primary level clinics are smaller clinics and are in rural and peri-urban areas. Secondary clinics are larger clinics located in urban areas; they provide a wider range of services and act as referral points for the primary level clinics. Tertiary clinics are hospitals located in large cities.

Seventeen sampling units of 19 participants will be selected at random from the list of eligible facilities in each strata using probability proportional to size based on mean monthly client flow in the past 3 months. If recruitment monitoring shows these clinics will not achieve full recruitment within the specified time-frame further clinics will be recruited. This will ensure 323 clients are recruited per strata, to allow between strata comparison, yielding a total sample of 969.

### Eligibility criteria

All women who meet the following inclusion criteria will be eligible for participation: (1) received MR with MVA or MRM from a participating clinic; (2) 18–49 years of age; (3) did not receive general anaesthesia for their MR procedure; (4) have a personal mobile telephone; (5) consent to receiving voice messages about family planning on their phone; (6) don’t intend to become pregnant within the next 6 months and (7) don’t intend to use (or for their partner to use) a permanent method of contraception in the next 6 months.

### Recruitment procedure

Participants will be recruited using the following steps, stages 1–4 are standard in the MR services provided at all MSB clinics and government facilities supported by Ipas. 1. MR is discussed with the client, the type of MR procedure is chosen and family planning is introduced, if a client wants an IUD fitted during the MVA procedure, this is agreed now. 2. The MVA procedure takes place or mifepristone (an oral pregnancy termination medication, also called RU486) is administered. 3. MVA clients can visit a recovery room. 4. Post-MR counselling is provided, including further discussion of family planning. Contraceptive method(s) are provided if desired. MVA clients can start any contraceptive method on the day of MR procedure, MRM clients can start any method apart from an IUD or female sterilization which can be provided after non-pregnancy has been established. 5. If the client is interested, she will be referred to a female research assistant (RA) who will explain the study and enrol her if she wishes. Alternatively, clients can return to the clinic within 2 days of their MR procedure for further information and enrolment. 6. Women who choose to participate will be asked to sign or thumbprint an informed consent form. Consent forms will also be signed by the interviewer and by a witness chosen by the client.

No financial incentives will be received for recruiting participants. Women participating in the study will receive 300 taka (approximately US$3.83 in February 2016) in cash at the time of enrolment as a token of appreciation for their time. They will receive an additional 100 taka for the short interview at 2 weeks post-MR and 200 taka for each of the longer follow-up interviews (at four and 12 months post-MR) using phone credit. To reduce loss to follow up, participants will be asked for any alternative phone numbers that can be used to contact them to conduct follow up interviews if we cannot reach them on their primary number.

To ensure the potential participants fully understands the intervention and to verify that it is not likely to put her at risk of harm, an example message will be played on the either the client’s or RA’s phone during recruitment (prior to randomisation) and the RA will explain how to use the interactive functions. After the message has been heard, the RA will ask the client whether she is happy to receive this type of message on her phone and what would happen if their husband, partner or someone else like her in-laws or other family member heard the message. If any concerns are raised the client will be advised not to participate and if necessary referred for further support.

### Randomisation

The allocation sequence will be generated remotely by a researcher at an independent research institute prior to the start of recruitment in Excel using the formula “=RANDBETWEEN(1, 2)”. The list will be password protected and only the independent researcher and a London based non-researcher (as a back-up) will have access to it. After each enrolment, RAs will send a unique participant ID and the participant’s phone number, family planning method and preferred message time slot to a technical officer based at the Marie Stopes Bangladesh (MSB) head office in Dhaka via a secure app. At the end of each day, the technical officer will email the IDs of new participants to the independent researcher for randomisation. The independent researcher will allocate participants to either the intervention or control group using the pre-generated randomisation list. On receiving the allocations from the independent researcher, the technical officer will initiate the intervention for those participants in the intervention group. This method will ensure allocations are concealed from the RAs recruiting clients and to service providers. Allocations will be concealed from RAs conducting the follow-up interviews during collection of data on the primary outcome and will also be concealed from one data analyst. Allocations are expected to be assigned within 2 days of enrolment. It will not be possible to blind participants to their allocation group. RAs conducting recruitment will not be un-blinded during the trial period.

### Intervention

The intervention was designed in 2015 based on 24 in-depth interviews with MR clients [23], literature reviews on barriers to and predictors of contraceptive use in Bangladesh, global evidence for the effectiveness of interventions delivered by mobile phone and behavioural theory [24]. Draft message content was tested with the target group. Full details of the development process will be reported elsewhere.

Each participant will be sent a series of 11 automated, interactive voice messages sent to their mobile phone over a 4-month period, starting within a week of the MR procedure. Messages will be sent weekly for the first 6 weeks and fortnightly for the following 8 weeks. The recorded messages, which are free to receive, start playing when the phone is answered and are delivered through a secure online platform from ‘I Am Digital’ in Dhaka. If the call is not answered it is re-sent after 30 min and again after another 30 min. At the end of each of the 11 messages, the recipient can press keys one to five to select one of the following options: (1) repeat the message; (2) listen to one or more of seven additional messages, one about each modern method of contraception (condoms, pills, injectable, implant, IUD, male and female sterilization); (3) speak to a call centre counsellor; (4) report that they are fine and don’t need further information today or (5) opt out from receiving further messages. At recruitment, the participant is asked to select one of five time slots for receiving the messages. The content of the 11 messages is tailored to the individual’s chosen method as follows: the method of contraception received at the clinic is used to allocate participants to one of six message groups: no method users, condom users, pill users, injectable users, implant users and IUD users. Seven core messages will be sent to all participants reminding them of the benefits of using contraception, addressing key barriers such as fear of infertility and addressing information gaps, particularly around LARC and permanent methods. The remaining four messages will be specific to the method group for example, pill users will receive the seven core messages plus four messages tailored to supporting pill use. For current contraceptive users, the tailored messages provide information and support for continuation and correct use of their chosen method, they also aim to promote safe-switching among women who are not happy with their method. The tailored messages sent to non-users are designed to encourage uptake of contraception. Women are reminded at recruitment and in the messages to inform the call centre counsellor if they switch contraceptive methods. The messages do not mention MR or refer to the participant’s clinic visit to prevent their MR status being disclosed should someone else answer or overhear the call.

The call centre is operated by three paramedics who have received training on MR, contraceptives and counselling. Women who contact the call centre are referred to MSB clinics or government facilities for services if needed.

Both control and intervention groups will receive post-MR counselling provided at the facility and information about the call centre which can be called at the cost of a standard call to a mobile phone. Women assigned to the control group will not receive any intervention messages.

### Data collection

Data collection tools will be developed in English then translated into and administered in Bangla. Where possible we will use or adapt existing tools including questions from the Bangladesh Demographic Health Survey [25]. On the first day of recruitment, data on the family planning services available at participating facilities will be collected from the facility manager or MR provider using a questionnaire administered by an RA (following informed consent). Study participants will be interviewed by the recruiting RA after enrolment to collect data on demographics (poverty level will be measured using the Progress out of Poverty Index (PPI)), reproductive history and intentions, the current MR and any family planning counselling and methods received, household decision making, experience of violence, type and frequency of mobile phone usage, contact phone number and preferred time of day to receive messages and to be contacted for follow up interviews.

Female RAs based at the MSB head office will administer follow-up questionnaires by telephone. The 2-week interview lasting approximately 5 min will ask about current contraceptive use. Changes in contraceptive method among intervention participant will be made in the online platform administering the messages. The 4-month and 12-month questionnaires will last approximately 15 min and will ask about marital status, fertility intentions, experience of violence, contraceptive use in the preceding months/weeks, any pregnancies or MR procedures, contraceptive discontinuation and reasons for stopping a method. Information on side effects will be collected where it led to discontinuation of a method. The 4 and 12 month follow up survey questions will be the same except that the time frame for some questions will differ and the 4-month survey will include measurement of hypothesised intermediate outcomes and questions to assess use of and satisfaction with the intervention and any related privacy concerns. Questions on the outcome will be asked at the start of the interview, prior to questions about the intervention.

All contraceptive use and non-use since baseline will be recorded using a modified version of the Demographic and Health Survey contraceptive calendar [25]. Participants will be asked at recruitment whether they consent to the study team accessing their clinic records to verify reports of MR procedures and LARC insertions or removals.

To ensure high participation in follow-up interviews we will collect a range of contact data for each participant and will ask participants to inform us if their contact details change. The reimbursements at each follow-up interview are also expected to increase participant retention.

#### Use of the intervention

Data on use of the intervention such as whether recorded voice messages are listened to and whether clients interact with the messages will be generated by the mHealth platform. Calls to the call centre will be monitored; call centre operators will complete a standard form for every caller to collect basic information on who the caller is (participant themselves, husband, friend etc.) and the reason or reasons for the call. A small number of calls will be audio recorded.

### Outcome measures

Primary and secondary outcomes and the methods of data collection are summarised in Table 1.

**Table 1:** Outcome variables and analysis methods

Outcome	Questionnaire	Outcome measure	Method of analysis
Primary			
LARC use	Baseline, 2-week, 4-month & 12-month	Self-reported (binary)	Logistic regression
Secondary			
Use of any modern contraceptive	Baseline, 2-week, 4-month & 12-month	Self-reported (binary)	Logistic regression
Pregnancy	4-month & 12-month	Self-reported (binary)	Logistic regression
MR	4-month & 12-month	Self-reported (binary)	Logistic regression
Discontinuation/ Method switching	2 week, 4-month & 12-month	Self-reported (#of times, continuous)	Log Rank test Kaplan Meier Curves
Experience of violence	4-month and 12 month	Self-reported (binary)	Logistic regression

#### Primary outcome

The primary outcome measure is use of a LARC (IUD or sub-dermal implant) at 4 months post-MR. Participants will be considered ‘LARC users’ if they report having a sub-dermal implant or IUD inserted at the 4-month follow up interview. The 4-month time point was selected to enable us to establish any effect of the intervention on continuation of the injection (effective for 3 months) and pills (clinics usually dispense 3 months-worth of pills).

#### Secondary outcomes

Secondary outcome measures include current use of any effective modern contraceptive method at 2 weeks, 4 and 12 months post MR. We define modern contraceptive methods according to the WHO as those associated with <10% 12 month pregnancy rates [13]. These will be measured as having had or having a partner who has had sterilization, currently have a sub-dermal implant or an IUD inserted, have received the injection in the last 3 months, currently using the contraceptive pill and reporting that they always or usually take the pill on time. Further secondary outcomes are self-reported pregnancy at the time of the four and 12 month interviews, having had an MR since enrolment into the study and having experienced violence since enrolment. Data from the contraceptive calendar will be used to estimate a point prevalence of contraceptive use at any given time and contraceptive discontinuation rates.

#### Hypothesized intermediate outcomes

At 4-months, data will be collected on the following hypothesized intermediate outcomes: satisfaction with family planning method, frequency of communication about family planning with husband and with in-laws, knowledge and attitudes towards family planning and self-efficacy to use contraception.

#### Dose

The effect of the dose of the intervention on the primary outcome will be examined using self-reported information on intervention use (listened to none, some or all of the messages), spoke to a call centre counsellor (yes/no).

### Sample size

**Fig. 1:**
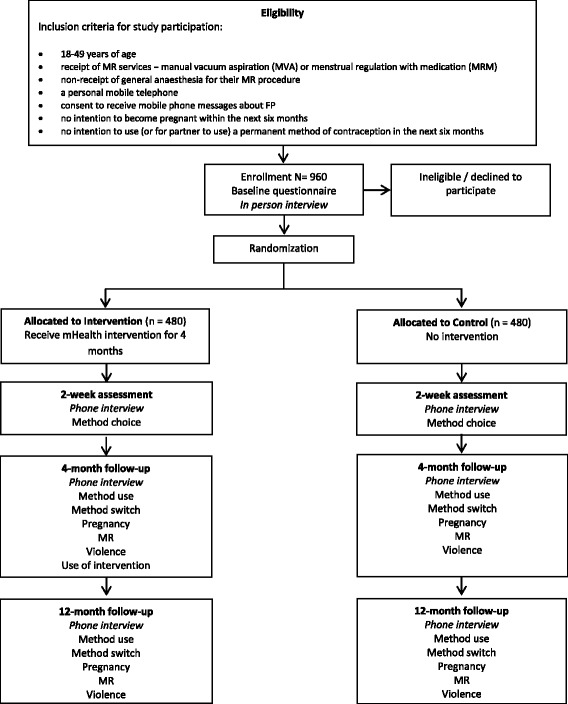
Study design and participant timeline

Sample size calculations were based on the primary outcome: LARC use 4 months after the MR procedure. Based on approximately 28,000 MR procedures conducted between November 2011 and June 2013 in government facilities supported by Ipas and approximately 16,500 in MSB clinics between October and December 2013, 7.0% of women are expected to select an IUD as their post-MR contraceptive method and 2.4% an implant, resulting in an expected 9.4% LARC uptake at baseline. A similar mHealth trial in Cambodia saw LARC use at 4 months increase from 9% to 29% with receipt of the intervention. Given the low cost of sending automated phone messages, a smaller increase may be programmatically important. To detect an absolute increase in LARC use of 7%, from 9.4% in the control group to 16.4% in the intervention group (a relative risk of 1.74), at four-month follow-up with a 0.05 significance level and 80% power, and assuming equal numbers of women in intervention and control groups the study needs a minimum of 714 participants. We increased this to a minimum of 960 to allow for 25% loss to follow-up (Fig. 1).

### Data management

The questionnaires will use anonymous ID numbers to identify respondents in the analysis. They will be composed of one digit to identify the clinic type (MSB/Ipas), followed by two digits for the facility code followed by a unique client identifier, composed of the day, month, staff member code and client number. An example of this could be M 01 2804 02 03. M indicates it is an MSB facility, 01 is the facility code. Two thousand eight hundred four is the date of the enrolment, 02 indicates the staff member code within the clinic and 03 indicates that it is the 3rd client enrolled on that day.

Study consent forms and baseline questionnaires will be photocopied at the facility. One copy will be posted to a central office for entry and storage, the other copy will be stored securely at the facility or in a locked location at the home of the research assistant until it can be collected in person by the research team. Two-week, 4 and 12 month questionnaires will be stored securely in a locked cabinet at MSB and the data entry company head offices in Dhaka. To protect women’s confidentiality, data will be recorded using numeric study identifiers, and all data (both hard copy and electronic) will be stored under the study identifiers and kept in locked file cabinets and password protected files, respectively. Data entry will be done in Microsoft Access by an external data entry specialist, and at least 10% will be double-entered. After entry, data will be checked for consistency and completeness. Data on contraceptive use will be cross-checked between time points, for example contraceptive calendar data collected at 4 months will be cross checked with baseline and 2-week data. Data will be checked for incompatible responses, for example being pregnant and using a method of contraception at follow-up*.* The data will be cleaned, analysed, and findings will be written up, by the principle investigators and researchers at Marie Stopes, Ipas and The London School of Hygiene and Tropical Medicine. Only the researchers conducting follow up surveys and those entering and checking data will have access to participant contact details. Audio recordings of calls to the call centre will be transcribed and translated into English.

### Training and monitoring

Research Assistants enrolling participants into the study and collecting baseline data will attend a 5-day training in Dhaka led by one international and one local investigator. Five supervisors will be trained to conduct supervision visits to ensure study protocols are being followed. Supervisors will be given a checklist to complete at each visit. Regular monitoring visits will be made by the study manager during enrolment and data collection to ensure study protocols are being followed and to identify any problems with recruitment or data collection. If necessary, further guidance or training will be provided to data collectors. Research Assistants conducting follow-up surveys will receive up to 5 days training (dependent on whether they have previous involvement in the study). Follow up data collection will be monitored by the study manager during daily de-briefs.

No data monitoring committee will be established for this trial as the likely risks to participants of receiving an mHealth intervention are known to be minimal. All adverse events reported to the study team during follow up surveys or via calls to the study or call centre numbers will be reported by the study manager to the principal investigator within 1 week of their occurrence. The response will be agreed with the other study investigators and where appropriate reported to the ethical committees.

The protocol and intervention were reviewed by an in-country advisory committee who will meet intermittently to review progress and give input. The committee includes the Director of Primary Health Care and Director of Continued Medical Education at the Directorate General of Health Services; the Director of Maternal and Child Health services and the Director of Information, Education and Motivation at the Directorate General of Family Planning; Executive Director, Reproductive Health Services, Training and Education Programme (a non-governmental organisation working in Government of Bangladesh Hospitals); Director, Bangladesh Association for Prevention of Septic Abortion; President, Obstetric and Gynaecological Society of Bangladesh; Country Director, Population Council; Chief Executive Officer, Bangladesh Centre for Communication Programme; Senior Director, Marie Stopes Bangladesh; Country Director, Ipas Bangladesh.

### Data analysis

We will adhere to the CONSORT guidelines for reporting RCT conduct and outcomes. Intention-to-treat principles will be used for primary outcome analysis, meaning all participants will be analysed according to the arm to which they were randomised. We will investigate the patterns of missing data, and will report any treatment group imbalances between those who do and do not complete the study.

The primary analysis, using multiple imputation to handle missing data (as detailed below), will be a logistic regression of 4-month LARC use on treatment and baseline use. For the primary analysis, we assume data are missing at random, given treatment group, 2-week and baseline LARC use, baseline socio-economic status (SES) and age. Under this assumption, multiple imputation of missing 4-month data will be performed separately for the two intervention groups, by full conditional specification, using the following variables: baseline, 2-week and 4-month LARC use, together with auxiliary variables, baseline SES and age. The multiple imputation procedure will also impute any missing baseline data (under the same assumption). One hundred imputed datasets will be created, and the imputation process will be checked for convergence and perfect prediction.

The primary analysis will be validated by an analysis under the slightly stronger assumption that data are missing at random, given treatment group, baseline LARC use, baseline SES and age. This can be achieved with a logistic regression of 4 month LARC use on baseline LARC use, intervention group, baseline SES and age. Robustness of inference from the primary analysis to the missing at random assumption will be explored using the “δ -method” for information-anchored sensitivity analysis [26].

Keeping the information in the data constant, this method allows us to explore the robustness of our conclusions about treatment to the missing at random assumption (MAR). In this study, MAR assumes that — among patients in the same treatment group, with similar baseline data and similar early follow-up — the odds of LARC use at the end is the same whether or not their data are missing. This assumption is untestable, so the “δ -method” explores how the conclusions vary as — still among patients in the same treatment group, with similar baseline data and similar early follow-up — the odds of LARC use at the end differs by δ between patients with observed and missing data.

Contraceptive discontinuation rates will be estimated over the one-year project period using Kaplan-Meier curves, overall and by family planning method. The log-rank test will be used to test for differences in discontinuation rates between methods. Table 1 summarizes the method of analysis for the primary and secondary outcomes. We will use Stata to conduct data analysis [24]. Data on use of the messages and details of calls made to the call centre will be analysed descriptively. Sub group analysis will be conducted to explore whether the intervention effect varies according to age, education, SES, type of MR procedure (medical vs surgical), experience of violence in the last year (yes/no), and on who makes decisions about whether the participant uses contraception (self/self and one or more other person/other person).

Transcripts from the calls to the call centre will be analysed qualitatively to examine the type of information and/or support the counsellors provide to enable a more detailed description of the intervention.

### Dissemination policy

Findings will be shared with stakeholders through formal reports and presentations at the local and national levels, and more broadly through peer-reviewed publications and international conference presentations. The researchers will present the findings to the advisory committee prior to wider dissemination.

Authorship eligibility will be dependent on substantial contributions to planning, implementing, analysing or drafting of findings. The de-identified dataset will be made publicly available following publication of trial results.

## Discussion

Closing knowledge gaps about effective methods of contraception is vital to supporting women’s choice and uptake of effective post-MR contraception. There have been few high quality evaluations of mHealth interventions designed to increase contraceptive use and continuation, particularly in low and middle income settings, and only one, MOTIF in Cambodia, has focused on a post-abortion population [22]. MOTIF also comprised an interactive voice message sent to mobile phones but used one standard message sent to all participants and relied heavily on call centre counsellors who provided personalised support and appointment bookings [16]. This one-to-one personalised support may be key to the intervention’s success but the cost of counselling could be a barrier to scale up. In the Bangladesh intervention counselling is still available however participants are first directed to automated content that aims to address some of the most commonly reported barriers to uptake and continuation of contraception in this context. It is important to explore whether automated content can reduce cost by replacing counsellor time but remain effective. This evaluation will provide much-needed information on the effectiveness of an mHealth to inform women about effective methods of contraception, and facilitate adoption of these methods in the post-MR period. mHealth has great promise as a low-cost intervention, and this trial will produce results that can inform programs and policies in Bangladesh and elsewhere.

## Abbreviations

IUD: Intrauterine device
LARC: Long-acting reversible contraceptive
mHealth: mobile-phone-based health
MR: Menstrual-regulation
MRM: Menstrual regulation with medicines
MSB: Marie Stopes Bangladesh
MSI: Marie Stopes International
MVA: Manual vacuum aspiration
PAC: Post-abortion care
RA: Research assistant
RCT: Randomised controlled trial
RHSTEP: Reproductive Health Services Training and Education Programme
SES: Socio-economic status
SMS: Short message service
